# Ampliação do uso de tecnologias para redução da malária por *Plasmodium vivax* na Amazônia brasileira: uma análise de impacto orçamentário

**DOI:** 10.1590/0102-311XPT044925

**Published:** 2026-03-16

**Authors:** Márcia Gisele Santos da Costa, Ivan Zimmermann, Ana Carolina Carioca da Costa, Ana Clara de Moraes, Márcia Pinto

**Affiliations:** 1 Núcleo de Avaliação de Tecnologias em Saúde, Instituto Nacional de Cardiologia, Rio de Janeiro, Brasil.; 2 Faculdade de Ciências da Saúde, Universidade de Brasília, Brasília, Brasil.; 3 Instituto Nacional de Saúde da Mulher, da Criança e do Adolescente Fernandes Figueira, Fundação Oswaldo Cruz, Rio de Janeiro, Brasil.; 4 Faculdade de Farmácia, Universidade Federal de Juiz de Fora, Juiz de Fora, Brasil.

**Keywords:** Financiamento da Assistência à Saúde, Anos de Vida Perdidos, Recursos em Saúde, Malária, Avaliação de Tecnologias de Saúde, Healthcare Financing, Years of Life Lost, Health Resources, Malaria, Health Technology Assessment, Financiación de la Atención de la Salud, Años de Vida Perdidos, Recursos en Salud, Malaria, Evaluación de Tecnologías de Salud

## Abstract

A tafenoquina e a primaquina são medicamentos utilizados no tratamento da malária por *Plasmodium vivax* e podem causar hemólise em pacientes com deficiência da enzima glicose-6-fosfato desidrogenase (G6PD). Recomenda-se a testagem da G6PD previamente à administração dos medicamentos. A tafenoquina e o teste quantitativo *point-of-care* G6PD estão incorporados ao Sistema Único de Saúde (SUS) para indivíduos maiores de 16 anos, mas não estão disponíveis para pacientes menores e de certas faixas de peso. A testagem para todos os pacientes elegíveis ao tratamento com primaquina também não é oferecida no SUS. O objetivo deste estudo foi calcular o impacto orçamentário incremental da incorporação da tafenoquina seguida da testagem da deficiência de G6PD para menores de 16 anos e por peso. Incluiu-se também a ampliação da testagem para os pacientes elegíveis ao tratamento com a primaquina. A perspectiva da análise é a do SUS para um horizonte de cinco anos. A população de referência foi a da Amazônia brasileira. Elaborou-se um modelo de impacto orçamentário que foi validado por gestores e especialistas em malária e assistência farmacêutica no SUS. O impacto orçamentário incremental foi de R$ 12,5 milhões. O modelo previu que a incorporação da tafenoquina reduziria os custos de hospitalização e de recorrências em cerca de 9% e 11%, respectivamente. O custo e o desperdício das tiras reagentes do teste impactaram nos resultados. As tecnologias avaliadas podem contribuir para a tomada de decisões mais seguras no tratamento da malária na Amazônia brasileira e parao planejamento, se as tecnologias em análise forem incorporadas.

## Abstract (en)

Tafenoquine and primaquine are drugs used in the treatment of malaria due to *Plasmodium vivax*, but can cause hemolysis in patients with glucose-6-phosphate dehydrogenase (G6PD) enzyme deficiency. Thus, G6PD testing is recommended prior to drug administration. Tafenoquine and the G6PD quantitative point-of-care test are incorporated into the Brazilian Unified National Health System (SUS) for individuals over 16 years of age, but are not available for younger patients or by weight range. Testing for all patients eligible for primaquine treatment is also not offered in the SUS. The aim of the present study was to calculate the incremental budgetary impact of the incorporation of tafenoquine and testing for G6PD deficiency in individuals under 16 years of age and by weight. The expansion of testing for patients eligible for treatment with primaquine was also considered. The perspective of the analysis is that of the SUS for a five-year horizon. The reference population was that of the Brazilian Amazon. A budget impact model was developed and validated by managers and specialists in malaria and pharmaceutical services in the SUS. The incremental budget impact was BRL 12.5 million. The model predicted that incorporating tafenoquine would reduce hospitalization and recurrence costs by about 9% and 11%, respectively. The cost and waste of the test reagent strips impacted the results. The technologies analyzed can contribute to safer decision-making in the treatment of malaria in the Brazilian Amazon and planning if the technologies under analysis are incorporated.

## Abstract (es)

La tafenoquina y la primaquina son medicamentos utilizados en el tratamiento de la malaria por *Plasmodium vivax* y pueden causar hemólisis en pacientes con deficiencia de la enzima glucosa-6-fosfato deshidrogenasa (G6PD). Se recomienda realizar una prueba de G6PD antes de administrar los medicamentos. La tafenoquina y la prueba cuantitativa de G6PD en el punto de atención están incorporadas al Sistema Único de Salud (SUS) para personas mayores de 16 años, pero no están disponibles para pacientes menores ni por rangos de peso. Las pruebas para todos los pacientes elegibles para el tratamiento con primaquina tampoco se ofrecen en el SUS. El objetivo de este estudio fue calcular el impacto presupuestario incremental de la incorporación de la tafenoquina seguida de pruebas de deficiencia de G6PD para menores de 16 años y por peso. También se incluyó la ampliación de las pruebas a los pacientes elegibles para el tratamiento con primaquina. La perspectiva del análisis es la del SUS para un horizonte de cinco años. La población de referencia fue la de la Amazonia brasileña. Se elaboró un modelo de impacto presupuestario que fue validado por gestores y especialistas en malaria y asistencia farmacéutica del SUS. El impacto presupuestario incremental fue de BRL 12,5 millones. El modelo previó que la incorporación de la tafenoquina reduciría los costes de hospitalización y de recurrencias en aproximadamente un 9% y un 11%, respectivamente. El costo y el desperdicio de las tiras reactivas de la prueba influyeron en los resultados. Las tecnologías evaluadas pueden contribuir a la toma de decisiones más seguras en el tratamiento de la malaria en la Amazonia brasileña y a la planificación si se incorporan las tecnologías analizadas.

## Introdução

A malária é uma doença prevenível e tratável e, nas últimas duas décadas, estima-se que 1,5 bilhão de casos e 7,6 milhões de mortes foram evitados globalmente ^1^. No entanto, aproximadamente, 2,5 bilhões de pessoas continuam sob o risco de transmissão por *Plasmodium vivax*, agente responsável por 75% dos casos ^2^. No Brasil, cerca 99% dos casos de malária estão concentrados na Amazônia brasileira, formada pelos estados do Acre, Amapá, Amazonas, Pará, Rondônia, Roraima, Tocantins, além de parte dos estados do Maranhão e do Mato Grosso ^3^. Em termos globais, a malária é uma das principais causas de mortes em crianças ^4^, e estudos realizados no Brasil mostraram que mais de 40% dos casos registrados de malária por *P. vivax* ocorrem nessa população, com a maior proporção observada em populações indígenas de até 10 anos ^5^.

As tecnologias avaliadas neste artigo incluem o uso do esquema com o medicamento tafenoquina após a testagem da deficiência de enzima glicose-6-fosfato desidrogenase (G6PD) em menores de 16 anos. Ensaios clínicos realizados recentemente demonstraram que a eficácia da tafenoquina é comparável ao esquema de baixa dose com a primaquina ^6^ ^,^ ^7^. Um dos principais cuidados na administração da tafenoquina em relação à segurança é o risco elevado de hemólise em pacientes com deficiência de G6PD ^7^ ^,^ ^8^ ^,^ ^9^. A atividade da enzima é sumarizada em três níveis, variando entre normal (até 70%), intermediária (30% a 70%) e deficiente (menor que 30%) ^10^. Tanto em situações de deficiência como de atividade intermediária, o uso de tafenoquina não é recomendado ^8^ ^,^ ^9^ ^,^ ^10^.

Embora apresente a grande vantagem de ser administrada em dose única, em comparação ao esquema medicamentoso com a primaquina, que tem a duração de 7 ou 14 dias ^6^ ^,^ ^7^, a tafenoquina requer acompanhamento proativo de profissionais de saúde, especialmente devido à sua meia-vida de aproximadamente 14 dias, o que significa que é metabolizada e eliminada lentamente pelo organismo em caso de reação hemolítica ^9^. Nesse sentido, para a cura radical, segura e eficaz de malária por *P. vivax*, deve-se considerar a implementação de testes rápidos que indiquem a deficiência de G6PD ^11^ ^,^ ^12^ ^,^ ^13^ ^,^ ^14^. A primaquina também pode causar hemólise em pessoas com essa deficiência e estima-se que, no Sistema Único de Saúde (SUS), ocorram cerca de 6 mil hospitalizações anuais por essa condição induzida pelo medicamento ^15^.

Há testes *point-of-care* disponíveis no mercado que utilizam um método quantitativo para detectar a deficiência de G6PD, permitindo decisões de tratamento mais seguras ^11^ ^,^ ^13^ ^,^ ^14^ ^,^ ^16^. Seu funcionamento ocorre pela utilização de biossensores e dispositivos eletrônicos portáteis com tiras reagentes ou cartuchos que medem simultaneamente a atividade de G6PD e os níveis de hemoglobina por meio de um analisador portátil operado por bateria. Após alguns minutos, o analisador fornece uma medição numérica da atividade de G6PD ^17^.

Ambas as tecnologias - tafenoquina e testagem da deficiência da enzima G6PD - foram recentemente aprovadas para a incorporação no SUS para indivíduos maiores de 16 anos, com base em evidências sobre a segurança e a eficácia do medicamento e sobre a adesão ao esquema medicamentoso com a tafenoquina conforme o resultado do teste ^18^.

Entre indivíduos de 2 a 15 anos, as evidências de um estudo multicêntrico de fase 2 demonstraram que a eficácia da tafenoquina para a recorrência por malária por *P. vivax* após quatro meses foi de 94,7%, em diferentes faixas de idade, peso e dosagens com comprimidos dispersíveis (50mg ou 150mg) ^19^. Ademais, essas dosagens se mostraram adequadas para os mesmos critérios de peso e idade ^19^. No entanto, nessas faixas etárias abaixo dos 16 anos, até recentemente, ainda não se observava a oferta da tafenoquina no SUS, diante de evidências científicas ainda insuficientes para garantir uma incorporação baseada na segurança e eficácia ^19^. Ademais, a testagem para os pacientes de todas as faixas etárias elegíveis à primaquina também não estava disponível ^3^.

Nesse contexto, o objetivo deste estudo foi estimar o impacto orçamentário da ampliação da testagem da deficiência da enzima G6PD seguida do regime com dose única de tafenoquina para menores de 16 anos ou com a primaquina, conforme faixas de peso e de idade. A análise foi realizada para os estados da Amazônia brasileira. O estudo justifica-se pela possibilidade de subsidiar as decisões de planejamento do gestor para a incorporação dessas tecnologias. Adotou-se a perspectiva do SUS, financiador no âmbito federal do diagnóstico e tratamento da malária por *P. vivax* no Brasil.

## Materiais e métodos

### Modelo de análise de impacto orçamentário

Foi elaborado um modelo analítico, do tipo árvore de decisão, estratificado por sexo, peso e idade e que incluiu parâmetros clínicos, de efetividade, de segurança e de custos do diagnóstico e tratamento da malária por *P. vivax*. O cenário de referência considerou a incorporação da tafenoquina (comprimidos dispersíveis de 50mg e 150mg) e do teste STANDARD G6PD (teste *point-of-care*), bem como a ampliação de uso deste teste para pacientes elegíveis ao regime com primaquina, durante um horizonte temporal de cinco anos. Este teste foi selecionado pois está disponível atualmente na rotina do SUS.

### População e cenários

A população elegível à tafenoquina foi definida como os indivíduos com menos de 16 anos, excluídas as gestantes e as crianças menores de seis meses de idade, conforme a recomendação do *Guia de Tratamento da Malária no Brasil* ^3^, e com diagnóstico de malária por *P. vivax*. Essa população foi estimada por demanda aferida a partir da série temporal de casos dos estados que fazem parte da Amazônia brasileira. O *Guia de Tratamento da Malária no Brasil* define os diferentes esquemas de tratamento da malária por *P. vivax* por peso e idade ^3^. A partir dos tratamentos recomendados, a população foi dividida em dois conjuntos de grupos etários e de peso. O primeiro corresponde aos não elegíveis à tafenoquina, definidos como indivíduos sem deficiência de G6PD, mas que se encontram em faixas de idade e de peso para as quais não existe indicação em bula ou evidência consolidada de segurança e eficácia do fármaco (5 a 9kg [6 a 11 meses], 10 a 14kg [1 a 3 anos], 15 a 24kg [4 a 8 anos], 25 a 34kg [9 a 11 anos], 35 a 49kg [12 a 14 anos] e 50 a 69kg [15 anos]). O segundo conjunto compreende os indivíduos elegíveis à tafenoquina, definidos como aqueles sem deficiência de G6PD e em faixas etárias e de peso nas quais o medicamento poderia ser utilizado segundo os cenários considerados no modelo (10 a 14kg [2 a 3 anos], 15 a 24kg [4 a 8 anos], 25 a 34kg [9 a 11 anos], 35 a 49kg [12 a 14 anos] e 50 a 69kg [15 anos]) ^3^.

Para os pacientes elegíveis à tafenoquina, considerou-se como cenário de referência a rotina atual do tratamento no SUS dos pacientes menores de 16 anos, sem a tafenoquina e sem a disponibilização do teste *point-of-care*. No cenário alternativo, haveria a incorporação do regime de tratamento com tafenoquina e rastreamento da atividade de G6PD, prevendo a probabilidade de ser detectada a deficiência ou não (atividade intermediária ou normal), conforme as faixas de peso e idade mencionadas.

Para os pacientes elegíveis à primaquina, o cenário de referência considerou a rotina atual do tratamento no SUS sem rastreamento de deficiência de G6PD e um cenário alternativo que propunha a ampliação da realização do teste *point-of-care*, com previsão da detecção da deficiência ou não (atividade intermediária ou normal) para todos os indivíduos com diagnóstico de malária por *P. vivax*.

### Previsão do número de casos diagnosticados de malária por *P. vivax* na Amazônia brasileira

Através de um modelo de predição com o horizonte temporal de cinco anos, obteve-se a previsão trimestral do número de casos confirmados de malária por *P. vivax* por sexo e idade para cada um dos nove estados. As séries temporais foram construídas a partir dos dados registrados no Sistema de Informação de Vigilância Epidemiológica da Malária (SIVEP-Malária) entre 2013 e 2023.

Na construção do modelo de predição, como primeira etapa, os bancos de dados foram divididos em dois conjuntos: treinamento e validação. Os dados de treinamento foram utilizados para o desenvolvimento do modelo de predição e compreenderam do 1º trimestre de 2013 ao último trimestre de 2021. Os dados de validação, por sua vez, compreenderam o período que variou do 1º trimestre de 2022 ao último trimestre de 2023. Foram consideradas quatro alternativas de modelos para fins de comparação de desempenho: o modelo de suavização exponencial (ETS, do inglês *exponential smoothing state space*), o modelo de Holt-Winters com dupla sazonalidade (DSHW, do inglês *double-seasonal Holt-Winters*), o modelo autorregressivo integrado de médias móveis (ARIMA, do inglês autoregressive integrated moving average) e o modelo TBATS (modelo de espaço de estado de suavização exponencial com transformação Box-Cox, erros ARMA, tendência e componentes sazonais). A qualidade do ajuste e a validade dos modelos propostos foram avaliadas com base no erro médio absoluto percentual (MAPE, do inglês mean absolute percentage error), tendo como referência os dados de validação (2022/1 a 2023/4) e nas análises do correlograma (ACF) e correlograma parcial (PACF) dos resíduos dos modelos. As previsões dos casos confirmados de malária por *P. vivax* por trimestre na população de estudo foram realizadas com base no modelo de melhor desempenho e as previsões anuais foram resultantes da soma das previsões trimestrais.

Como esta análise de impacto orçamentário foi realizada por peso do indivíduo, aplicou-se a proporção populacional por idade no total de casos para se conhecer o total da população elegível ^20^. Como etapa posterior, foram incluídos os custos diretos e as probabilidades relativas aos desfechos clínicos (deficiência de atividade de G6PD, recorrência e hospitalização devido à hemólise) e à acurácia do teste *point-of-care*, conforme a deficiência grave e a deficiência intermediária. Os softwares utilizados nas análises das previsões foram SPSS, versão 22 (https://www.ibm.com/), e R, versão 4.3.1 (http://www.r-project.org).

### Parâmetros do modelo

Os parâmetros adotados no modelo estão apresentados no Material Suplementar (Apêndice S1; https://cadernos.ensp.fiocruz.br/static//arquivo/suppl-e00044925_8727.pdf). Os casos de deficiência foram definidos para homens e mulheres com 30% ou menos de atividade da enzima G6PD e, para as mulheres, com atividade intermediária de G6PD que apresentavam limiar maior que 30% e menor ou igual a 70% ^11^. A frequência média anual de recorrência entre 5 e 60 dias no período de um ano de tratamento com a primaquina foi estimada em 1,134, com variação de 0,9206-1,3830 ^21^. Assumiu-se que número médio de recorrências anuais seria o mesmo para o esquema terapêutico com a primaquina e a tafenoquina.

O custo direto foi estimado a partir de dados literatura, dos preços de compra do Ministério da Saúde e dos valores disponíveis no Sistema de Gerenciamento da Tabela de Procedimentos, Medicamentos e OPM do SUS (SIGTAP) ^22^. Como o teste *point-of-care* está incorporado ao SUS, considerou-se que os insumos necessários para a sua realização (pilhas, luvas de procedimento, pipeta Pasteur, dentre outros) já estavam disponíveis na rede de atenção à saúde. Também, pelo mesmo motivo, a compra dos analisadores portáteis não foi considerada, nem dos controles para verificação da qualidade do teste. Para o custeio do diagnóstico com o teste *point-of-care*, incluiu-se somente o custo da tira reagente e de consultas ambulatoriais. Após relatos da experiência em campo da equipe responsável pelo estudo *Tafenoquine Roll-out Study* (TruST) ^23^, realizado em cidades da Amazônia brasileira, considerou-se uma margem adicional de 10% a 25% de tiras reagentes devido às possíveis perdas na execução do teste.

Para estimar o custo do tratamento farmacológico por peso e idade, utilizou-se como referência os esquemas preconizados no *Guia de Tratamento da Malária no Brasil* ^3^. Para este cálculo, foi elaborado um conjunto de possíveis regimes farmacológicos com a tafenoquina e a primaquina, por faixas de peso e de idade. Foi também adotado no modelo de impacto orçamentário o esquema medicamentoso preconizado como segunda opção, com artesunato e mefloquina ^3^. Esta inclusão foi necessária porque o tratamento de segunda linha com artesunato e mefloquina está indicado nos casos de recorrência de malária por *P. vivax* entre o quinto e o sexagésimo dia após o início da terapêutica. Esse padrão de recidiva pode sugerir falha na ação da cloroquina, da primaquina ou de ambos os fármacos. Os preços unitários dos medicamentos foram obtidos junto ao Ministério da Saúde e tiveram como referência a última compra realizada em 2024.

Calculou-se o custo médio de hospitalização dos pacientes com e sem diagnóstico de hemólise a partir da probabilidade estimada na literatura ^15^. A probabilidade condicional de hospitalização por hemólise foi aplicada somente para os indivíduos com deficiência da enzima G6PD. Optou-se por não aplicar a probabilidade bruta e sim a estimativa de probabilidade de hospitalização por hemólise, dado que o indivíduo tem um diagnóstico de malária por *P. vivax* e possui a deficiência da enzima G6PD. Da mesma forma, foi calculada a probabilidade de hospitalização sem hemólise em que o indivíduo tem um diagnóstico de malária por *P. vivax* e não possui a deficiência. A formulação matemática para o cálculo das probabilidades está apresentada no Material Suplementar (Apêndice S2; https://cadernos.ensp.fiocruz.br/static//arquivo/suppl-e00044925_8727.pdf).

Para a valoração das hospitalizações, utilizou-se os registros de internações hospitalares disponíveis no Sistema de Informações Hospitalares do SUS (SIH-SUS) durante o ano de 2024. Os intervalos de confiança (IC) foram estimados para ambas as probabilidades calculadas anteriormente. Foram selecionados dois grupos de pacientes com hospitalizações registradas conforme a Classificação Internacional de Doenças (10ª revisão): (i) pessoas com deficiência de G6PD, com hemólise, código B518 (“*Malária por Plasmodium vivax com outras complicações*”) e (ii) demais indivíduos hospitalizados, código B519 (“*Malária por Plasmodium vivax sem complicações*”). Os valores dos custos diretos são apresentados em reais (R$), se referem a 2024 e não houve ajuste inflacionário.

Como as compras dos analisadores e das tiras reagentes foram realizadas previamente pelo Ministério da Saúde, assumiu-se que a disponibilização de tais recursos para o diagnóstico e o tratamento da malária por *P. vivax* se iniciariam no ano 1 do horizonte temporal de cinco anos, garantindo a igualdade de acesso a toda população elegível dos estados da Amazônia brasileira.

O impacto orçamentário incremental foi calculado pela diferença entre o cenário de referência e o cenário alternativo, que se refere à ampliação do teste quantitativo para a população elegível ao esquema de primaquina e aos novos esquemas terapêuticos com a introdução da tafenoquina após a testagem, estratificados por sexo, faixas de peso e de idade. Os resultados do impacto orçamentário estão apresentados de acordo com seus principais componentes de custo direto (tratamento, hospitalizações, recorrências e oferta do teste *point-of-care*).

### Análise de sensibilidade

Uma análise de sensibilidade determinística foi realizada com o objetivo de identificar possíveis efeitos no resultado ocasionados pelo comportamento dos parâmetros ao longo do horizonte temporal. Um diagrama de tornado foi elaborado a partir da análise univariada dos parâmetros que mais poderiam impactar os resultados do modelo de impacto orçamentário.

### Processo de validação

Neste estudo, foram realizadas as validações interna (para garantir o rigor dos cálculos matemáticos) e aparente. A validação aparente objetivou verificar a aproximação do modelo proposto ao cenário real que se vislumbra caso as tecnologias sob análise fossem incorporadas ao SUS. Foram realizadas duas reuniões de consenso, no 2º semestre de 2024, entre a equipe da pesquisa e os gestores responsáveis pelo financiamento e planejamento da atenção à saúde em malária, bem como pela organização do processo de compra e distribuição de tecnologias no SUS. Um roteiro foi aplicado com perguntas sobre a viabilidade das tecnologias serem incorporadas no contexto atual da atenção à saúde na Amazônia brasileira. O modelo de impacto orçamentário foi apresentado a fim de garantir a transparência de todo o seu processo de desenvolvimento, bem como para validar parâmetros e pressupostos.

### Considerações éticas

O estudo de impacto orçamentário foi conduzido sem submissão prévia ao Comitê de Ética em Pesquisa (CEP) por se enquadrar em situação de dispensa de avaliação do sistema CEP/CONEP de acordo com a *Resolução CNS nº 510/2016*. Ressalta-se que as estimativas foram obtidas a partir de dados secundários públicos e não identificáveis.

## Resultados

### Previsão do número de casos de malária por *P. vivax* por estado da Amazônia brasileira

**Figura 1:**
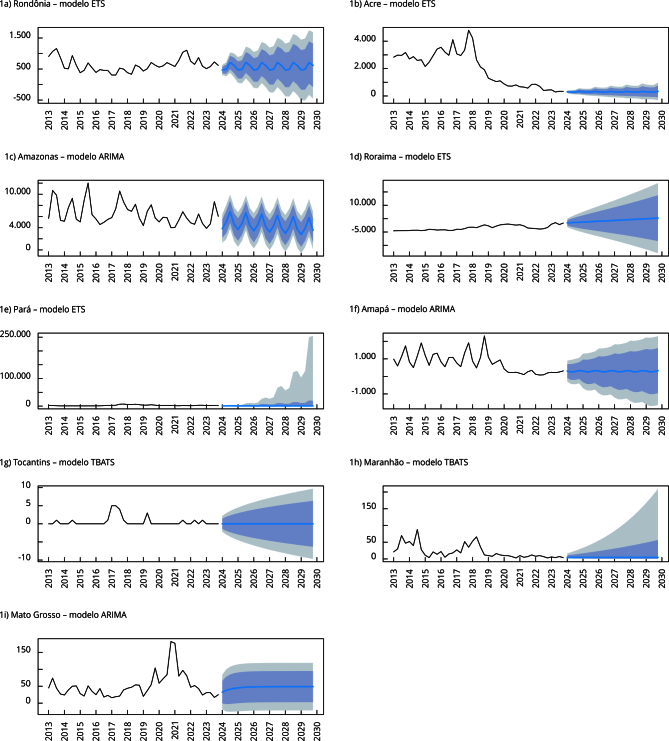
Número de casos confirmados trimestralmente de malária por *Plasmodium vivax* observado e projeções em indivíduos com seis meses ou mais e menores de 16 anos nos estados de Rondônia, Acre, Amazonas, Roraima, Pará, Amapá, Tocantins, Maranhão e Mato Grosso, Brasil, 1º trimestre de 2013 a 4º trimestre de 2029.

ARIMA: modelo autorregressivo integrado de médias móveis; DSHW: modelo de Holt-Winters com dupla sazonalidade; ETS: modelo de suavização exponencial; TBATS: T (sazonalidade trigonométrica), B (transformação Box-Cox), A (erros ARIMA), T (tendência), S (componentes sazonais).

Na análise da série temporal do número de casos de malária por *P. vivax* projetados ao longo de cinco anos, pôde-se observar claramente um padrão sazonal (Figura 1). A amplitude dos ICs aumentou à medida que o horizonte de previsão alcançou o ano 5 devido à incerteza associada. No período de cinco anos, seriam realizados 213.627 testes *point-of-care*. Os estados do Amazonas (88.465) e Roraima (89.133) demandariam anualmente a maior quantidade de testes ou 83% de todo o quantitativo (Tabela 1).

**Tabela 1:** Projeção de casos de malária por *Plasmodium vivax*, em cinco anos, considerando indivíduos com seis meses ou mais e menores de 16 anos na Amazônia brasileira.

Estado	Ano 1	Ano 2	Ano 3	Ano 4	Ano 5	Total
Rondônia	2.289	2.289	2.289	2.289	2.289	11.445
Acre	1.197	1.197	1.197	1.197	1.197	5.985
Amazonas	19.469	18.581	17.693	16.805	15.917	88.465
Roraima	15.358	16.592	17.827	19.061	20.295	89.133
Pará	2.693	2.361	2.237	2.189	2.169	11.649
Amapá	1.175	1.176	1.177	1.177	1.177	5.882
Tocantins	1	1	1	1	1	5
Maranhão	19	19	19	19	19	95
Mato Grosso	187	194	195	196	196	968
Total	42.388	42.410	42.635	42.934	43.260	213.627

Fonte: elaboração própria, a partir do Sistema de Informação de Vigilância Epidemiológica da Malária (SIVEP-Malária).

No cenário de referência, em que não haveria ampliação da testagem da deficiência de G6PD, 205.200 e 8.427 pessoas, com atividade intermediária e com deficiência de G6PD, receberiam a primaquina em dose normal e primaquina em baixa dose, respectivamente. Com a ampliação da testagem, 200.764 pacientes receberiam a primaquina em dose normal, enquanto 12.863 receberiam a primaquina em baixa dose (Tabela 2). Um total de 213.627 indivíduos seriam atendidos, dos quais 85% seriam elegíveis ao regime terapêutico com a tafenoquina. Ainda permaneceriam 20.149 e 12.863 pessoas nos regimes de primaquina (tratamento normal) e de baixa dose, respectivamente (Tabela 2).

**Tabela 2:** Projeção do número de pessoas atendidas de acordo com cenários de incorporação: sem ampliação e com ampliação da testagem de deficiência da enzima glicose-6-fosfato desidrogenase (G6PD) para pacientes elegíveis ao regime com primaquina na Amazônia brasileira.

Período	Pessoas atendidas					
	Sem ampliação da testagem de deficiência de G6PD			Com ampliação da testagem de deficiência de G6PD		
	Primaquina em dose normal	Primaquina em baixa dose	Total	Primaquina em dose normal	Primaquina em baixa dose	Total
Ano 1	40.716	1.672	42.388	39.836	2.552	42.388
Ano 2	40.737	1.673	42.410	39.856	2.554	42.410
Ano 3	40.953	1.682	42.635	40.068	2.567	42.635
Ano 4	41.240	1.694	42.934	40.349	2.585	42.934
Ano 5	41.553	1.707	43.260	40.655	2.605	43.260
Total	205.200	8.427	213.627	200.764	12.863	213.627

Fonte: elaboração própria, a partir do Sistema de Informação de Vigilância Epidemiológica da Malária (SIVEP-Malária).

O detalhamento dos custos mostrou que, no cenário de referência, em que não há ampliação da testagem e do uso da tafenoquina para menores de 16 anos, o custo total no período de cinco anos seria de aproximadamente R$ 4 milhões. O principal direcionador de custos foram as hospitalizações e o tratamento com a primaquina (74%). No cenário alternativo, com a ampliação do uso de ambas as tecnologias (testagem G6PD e tafenoquina), o custo total mais que quadriplicou (R$ 16,6 milhões) e o teste *point-of-care* foi responsável pela metade do custo total. Observou-se uma redução no custo das hospitalizações neste cenário de, aproximadamente, 9%, bem como de 11% nas recorrências ao longo de cinco anos. O detalhamento dos custos do tratamento com a primaquina e a tafenoquina, da ampliação da testagem, das hospitalizações e das recorrências para o cenário de referência e para o cenário alternativo está apresentado no Material Suplementar (Apêndice S3; https://cadernos.ensp.fiocruz.br/static//arquivo/suppl-e00044925_8727.pdf).

O impacto orçamentário incremental estimado foi de aproximadamente R$ 12,5 milhões no decorrer do período estudado. A ampliação da testagem e do uso da tafenoquina totalizariam R$ 8,1 milhões (65%) e R$ 4,4 milhões (35%), respectivamente. Estimou-se em média a necessidade de R$ 2,5 milhões ao ano para ampliar a oferta das duas tecnologias (Tabela 3).

**Tabela 3:** Impacto orçamentário incremental da ampliação do uso da tafenoquina e da testagem de deficiência da enzima glicose-6-fosfato desidrogenase (G6PD) na Amazônia brasileira.

Período	Custo incremental (R$)		
	Com ampliação de testagem de deficiência de G6PD	Com ampliação de uso de tafenoquina	Total
Ano 1	1.603.569,65	870.876,15	2.474.445,80
Ano 2	1.604.401,93	871.328,15	2.475.730,08
Ano 3	1.612.913,85	875.950,85	2.488.864,70
Ano 4	1.624.225,24	882.093,91	2.506.319,15
Ano 5	1.636.558,06	888.791,69	2.525.349,75
Total	8.081.668,73	4.389.040,74	12.470.709,48

Fonte: elaboração própria, a partir do Sistema de Informação de Vigilância Epidemiológica da Malária (SIVEP-Malária).

O processo de validação aparente realizado com gestores e técnicos que atuavam no planejamento e ações de controle da malária e da compra e distribuição de insumos para o SUS permitiu que o desenvolvimento do modelo fosse baseado nas melhores práticas. As reuniões de consenso apoiaram todo o percurso do presente estudo e, de forma convergente, permitiram que participantes discutissem a estrutura do modelo e seus cenários, os parâmetros e os pressupostos que mais se aproximassem da realidade em um contexto de incorporação das tecnologias na Amazônia brasileira.

**Figura 2:**
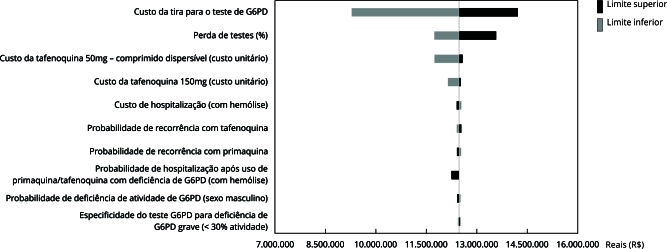
Análise em tornado.

A análise de sensibilidade indicou o custo da tira reagente do teste *point-of-care* como o principal modificador dos resultados quando comparado a todas as demais variáveis de relevância para o modelo. Em seguida, a perda de testes e o custo da tafenoquina (50mg com comprimidos dispersíveis) foram os parâmetros que mais influenciaram os resultados (Figura 2).

G6PD: enzima glicose-6-fosfato desidrogenase.

## Discussão

O impacto orçamentário incremental da incorporação do teste *point-of-care* seguido da tafenoquina e da ampliação da testagem aos pacientes elegíveis ao tratamento com primaquina seria de R$ 12 milhões em cinco anos, sob a perspectiva do SUS. Em 2023, a tafenoquina e a testagem da deficiência de G6PD foram incorporadas para indivíduos acima de 16 anos. A incorporação foi apoiada em evidências geradas em estudos nacionais e internacionais que reforçaram a efetividade dos testes quantitativos, a redução das recaídas em seis meses em comparação com a primaquina, bem como a adesão ao tratamento ^6^ ^,^ ^7^ ^,^ ^11^ ^,^ ^23^.

Os estudos de performance e de avaliação econômica indicaram que a incorporação da testagem da deficiência de G6PD é custo-efetiva para o SUS, bem como a incorporação da tafenoquina ^10^. Inclusive, o esquema com as duas tecnologias mostrou-se custo-efetivo nos cenários em que crianças com mais de seis meses poderiam ser tratadas com a formulação pediátrica e nas regiões onde a aderência à primaquina é baixa ^10^. Em outros países, entre os pacientes incluídos no tratamento medicamentoso com a dose única da tafenoquina, houve melhora na adesão quando comparada aos pacientes incluídos no esquema da primaquina, sendo destacadamente vantajosa em áreas onde o acesso aos serviços de saúde era limitado ^10^ ^,^ ^23^ ^,^ ^24^ ^,^ ^25^ ^,^ ^26^. A literatura também tem demonstrado evidências favoráveis à incorporação de ambas as tecnologias devido à redução do custo das complicações por hemólise e das recorrências, além de evitar custos indiretos por perda de produtividade para o trabalho ^25^ ^,^ ^26^ ^,^ ^27^. No presente estudo, observou-se uma redução de 9% e 11%, no custo das hospitalizações e das recorrências, respectivamente.

Os resultados da custo-efetividade foram favoráveis à incorporação da testagem *point-of-care* da deficiência de G6PD ^10^, mas são as análises de impacto orçamentário que orientam o planejamento e a decisão de compra do tomador de decisão ao prever o montante de recursos a ser disponibilizado para garantir a oferta das tecnologias. Um estudo de impacto orçamentário da incorporação de um programa de *screening* com G6PD no Laos mostrou que nos diferentes cenários estudados, o custo foi elevado, alcançando cerca de USD 450 mil (R$ 2,7 milhões ao ano) ^25^. A análise concluiu que o valor era economicamente inviável para a realidade daquele país. No caso do SUS, o impacto incremental se aproximou de tais valores (R$ 12,5 milhões) em cinco anos, um montante de recursos a ser considerado no orçamento das ações voltadas à redução da morbimortalidade por malária por *P. vivax*. Reconhece-se que a eficiência na incorporação das tecnologias em análise envolve outros aspectos tão importantes quanto os econômicos, como, por exemplo, aqueles relacionados à efetividade da assistência e ao acesso. Nesse sentido, a adesão dos profissionais de saúde tem se mostrado favorável na administração da tafenoquina e na realização do teste ^7^ ^,^ ^23^. Ademais, a testagem permite o rastreio adequado de indivíduos com deficiência de G6PD e, com a incorporação da tafenoquina pediátrica, será ampliada a oferta de medicamentos às populações de baixo peso, conforme indicado na bula aprovada recentemente pela Agência Nacional de Vigilância Sanitária (Anvisa) ^28^.

Apesar das vantagens, os resultados aqui apresentados devem ser interpretados à luz da lógica de cofinanciamento do SUS no processo de incorporação de tecnologias e das contrapartidas dos três entes federativos sob o ponto de vista da gestão tripartite. Importa ressaltar que os analisadores e os reagentes foram adquiridos e distribuídos previamente pelo Ministério da Saúde para os estados da Amazônia brasileira. Isso demonstra, no caso específico desta incorporação, um avanço nas discussões sobre a redistribuição de responsabilidades entre os entes federativos, bem como sobre os limites do financiamento de cada esfera. Neste estudo, ainda que o impacto orçamentário tenha indicado uma possível economia de recursos, como a redução de hospitalizações, a sustentabilidade orçamentária não se restringe apenas à essa economia projetada no cenário alternativo, mas depende sobretudo da disponibilidade e da alocação dos recursos nas esferas de governo tanto na assistência farmacêutica quanto no planejamento, compra e distribuição dos testes. Nesse sentido, a necessidade de alinhamento entre União, estados e municípios deve ser um processo contínuo na política de incorporação de tecnologias no SUS, fundamental para garantir a efetividade do tratamento, o acesso e a maximização dos benefícios de saúde para a sociedade.

Em nosso estudo, a análise de sensibilidade realizada mostrou que as tiras reagentes e a perda de testes foram os principais parâmetros que influenciaram o modelo. Tal resultado indica que o monitoramento do desperdício deve ser contínuo pela possibilidade de redução do custo total desses insumos. Nesse contexto, como o teste *point-of-care* já está incorporado ao SUS desde 2023, a curva de aprendizagem dos profissionais de saúde possivelmente já foi superada e espera-se que, no médio prazo, tal perda possa ser reduzida. É necessário considerar as questões de armazenagem, já que as tiras não podem ser mantidas em geladeira, devem ser preservadas na temperatura de 2ºC a 30ºC e conservadas na embalagem selada até o momento do uso. Ainda que o teste possa ser realizado em temperaturas que variam de 15ºC a 40ºC, este é um ponto de atenção dada as atuais condições climáticas observadas no Brasil ^29^ ^,^ ^30^.

Na estimativa da população, para alguns estados, especialmente para o Tocantins e o Maranhão, a série de casos confirmados de malária por *P.vivax* não apresentou uma tendência de crescimento ou de redução bem estabelecida entre 2013 e 2023. Isso ocorreu devido ao reduzido número de casos de malária por *P. vivax* nesses estados. Em razão disso, o modelo DSHW não foi aplicado aos estados do Tocantins e do Maranhão. Ademais, os resultados dos valores preditos de casos ao longo do horizonte temporal apresentaram uma linha constante naqueles estados, o que limitou a análise de previsão de casos nos cinco anos do horizonte temporal. Apesar dessa limitação, a apresentação dos resultados da população elegível por estado, por ano e ainda por trimestre teve como objetivo gerar mais evidências para o gestor na sua tomada de decisão, destacadamente, no planejamento e na gestão das tecnologias e dos recursos a serem investidos.

A literatura indica que é uma boa prática em estudos de impacto orçamentário na área da saúde, a realização da validação aparente com os tomadores de decisão e a validação interna ^31^. Assim, para que um modelo seja útil aos tomadores de decisão, além da confiança nos resultados, eles precisam saber com quanta precisão o modelo prediz os resultados de interesse ^31^. Nesse sentido, a realização da validação aparente foi um momento importante neste estudo, propiciando o suporte da equipe de gestores e técnicos em relação à condução do modelo de impacto orçamentário. Ademais, as discussões permitiram garantir a transparência no processo de desenvolvimento, como a definição dos cenários, dos parâmetros e o cálculo dos custos. Nas duas reuniões ocorridas em 2024, estabeleceu-se um consenso entre toda a equipe em relação às melhores evidências para se alcançar os objetivos propostos nesta análise.

Finalmente, a inclusão da tafenoquina em dose única para menores de 16 anos, com a possibilidade de ajuste da dose por peso, pode permitir a disponibilização de um novo esquema medicamentoso para crianças, incluindo aquelas com problemas de desnutrição, bem como para os adolescentes e adultos nestas mesmas condições. Há anos, os estudos realizados na Amazônia brasileira reportam a incidência de anemia e os índices peso, estatura e de massa corporal conforme a idade como preocupantes ^32^ ^,^ ^33^. Nesse sentido, é de extrema importância que evidências robustas sejam geradas para a incorporação de tecnologias em saúde baseadas na segurança, efetividade, eficácia, viabilidade econômica e viabilidade operacional, aqui incluída a logística e a disponibilização ágil de recursos e insumos aos profissionais de saúde envolvidos na assistência à malária por *P. vivax*. Ações para que todos esses aspectos científicos e organizacionais convirjam são um desafio considerando os permanentes problemas observados na Amazônia brasileira. Espera-se que este estudo possa contribuir para que o Brasil alcance as metas estabelecidas para a eliminação dos casos de malária por *P. vivax*.

## Data Availability

Os dados de pesquisa estão disponíveis mediante solicitação à autora de correspondência.

## Material Suplementar

Material Suplementar
