# Supplementary material for: Expansion of the use of technologies to reduce cases of malaria due to Plasmodium vivax in the Brazilian Amazon: a budget impact analysis
Source: Cad Saude Publica. 2026 Mar 16;42:e00044925. [Article in Portuguese] doi: 10.1590/0102-311XPT044925 (PMC12995294; doi:10.1590/0102-311XPT044925)
Supplement: Material Suplementar [file 1678-4464-csp-42-PT044925-s.pdf]

MATERIAL SUPLEMENTAR

APÊNDICE S1

Tabela S1 Parâmetros do modelo de análise de impacto orçamentário para a ampliação do uso da tafenoquina e do teste *point-of-care* para pacientes elegíveis ao regime com primaquina, conforme faixas de peso e de idade.

**Descrição do parâmetro**	**Estimativa pontual**[§]	**Limite Inferior**	**Limite Superior**	**Fonte**
Probabilidade da deficiência atividade de G6PD (sexo feminino)	0,42%	0,13%	1,02%	1
Probabilidade da atividade intermediária de G6PD (sexo feminino)	3,69%	2,63%	5,04%	1
Probabilidade da deficiência atividade de G6PD (sexo masculino)	6,85%	5,24%	8,78%	1
Probabilidade de recorrência com primaquina em dose baixa	17,90%	13,20%	22,30%	2
Probabilidade de recorrência com primaquina	26,60%	25,00%	28,10%	2
Probabilidade de recorrência com tafenoquina	24,20%	22,40%	26,00%	2
Número médio de recorrências em 1 ano	1,1340	92,06%	1,3830	3
Especificidade do teste G6PD para deficiência de G6PD grave (<30% atividade)	97,80%	97,00%	98,50%	4
Sensibilidade do teste para deficiência de G6PD grave (<30% atividade)	100,00%	93,80%	100,00%	4
Sensibilidade do teste G6PD intermediário (<70% no sexo feminino)	94,30%	80,80%	99,30%	4
Probabilidade de hospitalização após uso de PQ/TQ com deficiência de G6PD (com hemólise)	4,60%	8,07%	11,86%	Estimado a partir de 1
Probabilidade de hospitalização após uso de PQ/TQ sem deficiência de G6PD (sem hemólise)	2,23%	1,97%	2,31%	Estimado a partir de 1
Probabilidade do sexo masculino	100,00%	54,66%	54,91%	Sivep-Malária, 2023
Custo da tafenoquina 150mg (custo unitário)	R$ 16,20	R$ 12,93	R$ 16,67	CMED - PMVG de 18%
Custo da tafenoquina 50mg - comprimido dispersível (custo unitário)	R$ 10,76	R$ 8,59	R$ 11,07	CMED - PMVG 18%
Custo da tira para o teste de G6PD	R$ 34,42	R$ 20,85	R$ 41,85	Distribuidor do equipamento no Brasil
**Perda de testes (%)**	**10%**	**0%**	**25%**	**Pressuposto**
Custo da hospitalização (com hemólise)	R$ 385,19	R$ 204,83	R$ 565,56	SIH-SUS, 2023
Custo da hospitalização (sem hemólise)	R$ 297,99	R$ 228,75	R$ 367,23	SIH-SUS, 2023
Custo da cloroquina 150 mg (custo unitário)	R$ 0,41	-	-	Ministério da Saúde (último preço de aquisição, 2024)
Custo da primaquina 5 mg (custo unitário)	R$ 0,30	-	-	Ministério da Saúde (último preço de aquisição, 2024)
Custo da primaquina 15 mg (custo unitário)	R$ 0,50	-	-	Ministério da Saúde (último preço de aquisição, 2024)

Custo do artesunato 25mg + mefloquina 50mg (custo unitário)	R$ 0,19	-	-	Ministério da Saúde (último preço de aquisição, 2024)
Custo do artesunato 100mg + mefloquina 220mg (custo unitário)	R$ 2,38	-	-	Ministério da Saúde (último preço de aquisição, 2024)
Custo de insumos por cada teste (luva, pipeta pasteur e filme)	R$ 1,13	-	-	Banco de Preços em Saúde - base SIASG
Custo das Tiras para o teste de G6PD (preço e adição de perdas)	R$ 37,86	-	-	Distribuidor do equipamento
**Custo do tratamento medicamentoso sem tafenoquina (sem deficiência de G6PD) ***				
Peso 5-9 Kg (6 a 11 meses)	R$ 2,64	-	-	5
Peso 10-14 Kg (1 a 3 anos)	R$ 5,39	-	-	5
Peso 15-24 Kg (4 a 8 anos)	R$ 5,15	-	-	5

* Refere-se ao custo estimado conforme os esquemas de tratamento preconizados pelo Guia de tratamento da malária no Brasil, tratamentos propostos para o esquema de tafenoquina e nos preços obtidos junto ao Ministério da Saúde.

SIVEP – Malária: Sistema de Informações de Vigilância Epidemiológica - Notificação de casos de Malária.

CMED – PMVG: Câmara de Regulação do Mercado de Medicamentos – Preço Máximo de Venda ao Governo.

SIH-SUS: Sistema de Informações Hospitalares do Sistema Único de Saúde.

SIASG: Sistema Integrado de Administração de Serviços Gerais.

Referências

1. Zobrist S, Brito M, Garbin E, Monteiro WM, Clementino Freitas S, Macedo M, et al. Evaluation of a point-of-care diagnostic to identify glucose-6-phosphate dehydrogenase deficiency in Brazil. PLOS Neglected Tropical Diseases. 2021;15(8):e0009649. doi:10.1371/journal.pntd.0009649.

2. Brito M, Rufatto R, Murta, M, Sampaio V, Balieiro P, et alOperational feasibility of Plasmodium vivax radical cure with tafenoquine or primaquine following point-of-care, quantitative glucose-6-phosphate dehydrogenase testing in the Brazilian Amazon: a real-life retrospective analysis Lancet Glob Health. 2024; 12:e467-e477

3. Taylor AR, Watson JÁ, Chu CS, Puaprasert K, Duanguppama J et al. Resolving the cause of recurrent Plasmodium vivax malaria probabilistically. Nature Communications. 2019; 10:5595.

4. Zobrist S, Brito M, Garbin E, Monteiro WM, Clementino Freitas S, Macedo M, et al. Evaluation of a point-of-care diagnostic to identify glucose-6-phosphate dehydrogenase deficiency in Brazil. PLOS Neglected Tropical Diseases. 2021;15(8):e0009649. doi:10.1371/journal.pntd.0009649.

5. Brasil. Ministério da Saúde. Secretaria de Vigilância em Saúde. Departamento de Imunização Doenças Transmissíveis. Guia de tratamento da malária no Brasil / Ministério da Saúde, Secretaria de Vigilância em Saúde, Departamento de Imunização e Doenças Transmissíveis – 2. ed. atual. - Brasília: Ministério da Saúde, 2021.

APÊNDICE S2

Cálculo das probabilidades de hospitalização por hemólise e sem hemólise para estimar o custo médio

- Probabilidade de hospitalização por hemólise: Temos: $ph = a(b \times fd)$

Onde,

ph: probabilidade de hospitalização por hemólise.

a: número de casos com hemólise hospitalizados.

b: número de casos de Plasmodium vivax tratados.

fd: fração de indivíduos com deficiência de G6PD.

Considerando os dados de Brito-Souza *et al.* (2019)[1] e uma probabilidade de deficiência de G6PD de 3,40%, relatada por Zobrist *et al*. (2021)[2], temos:

$pa = 94(28095 * 0{,}034)$

$pa \cong \mathbf{9{,}84}\%$

- Probabilidade de hospitalização sem hemólise: Temos: $pb = c(b \times (1 - fd))$

Onde,

pb: probabilidade de hospitalização sem hemólise

c: número de casos sem hemólise hospitalizados

b: número de casos de Plasmodium vivax tratados

fd: fração de indivíduos com deficiência de G6PD

Havia um total 672 hospitalizações relatados por Brito-Souza *et al*. (2019) e que apenas 94 destas seriam devido a hemólise, as demais (578) seriam hospitalização sem curso de hemólise.

Assim:

$pb = 578(28095 \times (1 - 0{,}034))$

$pb \cong 578(28095 * 0{,}034)$

$pb \cong \mathbf{2{,}13}\%$

Referências

1. Brito-Sousa JD, Santos TC, Avalos S, Fontecha G, Melo GC et al. Clinical Spectrum of Primaquine-induced Hemolysis in Glucose-6-Phosphate Dehydrogenase Deficiency: A 9-Year Hospitalization-based Study From the Brazilian Amazon. Clin Infect Dis. 2019 Sep 27;69(8):1440-1442.

2. Zobrist S, Brito M, Garbin E, Monteiro WM, Clementino Freitas S, Macedo M, et al. Evaluation of a point-of-care diagnostic to identify glucose-6-phosphate dehydrogenase deficiency in Brazil. PLOS Neglected Tropical Diseases. 2021;15(8):e0009649. doi:10.1371/journal.pntd.0009649.

APÊNDICE S3

Detalhamento dos custos

Tabela S2 Custos do cenário de referência, Amazônia Brasileira. Ano 1 ao ano 5 (em R$ de 2023).

**Cenário de Referência**				
**Período**	**Tratamento com Primaquina**	**Hospitalizações**	**Tratamento de recorrências**	**Total**
Ano 1	301.877,50	302.936,54	211.456,53	816.270,57
Ano 2	302.034,18	303.093,77	211.566,28	816.694,23
Ano 3	303.636,58	304.701,79	212.688,71	821.027,08
Ano 4	305.765,99	306.838,67	214.180,30	826.784,96
Ano 5	308.087,68	309.168,51	215.806,59	833.062,78
**Total**	**1.521.401,93**	**1.526.739,28**	**1.065.698,42**	**4.113.839,62**

Fonte: Elaboração própria.

Tabela S3 Custos do cenário alternativo, Amazônia Brasileira. Ano 1 ao ano 5 (em R$ de 2023).

	**Teste de G6PD**	**Tratamento com Primaquina**	**Tratamento com Tafenoquina**	**Hospitalizações**	**Tratamento de recorrências**	**Total**
Ano 1	1.652.924	138.060	1.031.310	278.127	190.295	3.290.716
Ano 2	1.653.782	138.131	1.031.845	278.272	190.394	3.292.424
Ano 3	1.662.556	138.864	1.037.319	279.748	191.404	3.309.892
Ano 4	1.674.216	139.838	1.044.594	281.710	192.747	3.333.104
Ano 5	1.686.928	140.900	1.052.526	283.849	194.210	3.358.413
**Total**	**8.330.406**	**695.793**	**5.197.594**	**1.401.706**	**959.050**	**16.584.549**

Fonte: Elaboração própria.
